# New Treatment of Carbuncle

**Published:** 1896-12

**Authors:** 


New Treatment of Carbuncle.—Readers will be interested in the paper of Dr. T. C. Osborn in this issue.

The doctor's claim to priority in aborting small-pox by local means having been disputed, in this paper, after referring to that subject, and pointing out that he used an aqueous solution, which, he insists, is not so irritating as the alcoholic solution recommended by other claimants to the discovery that germs can be destroyed *in situ*—the writer then calls attention to his method of dealing with carbuncle, which, he says has been eminently satisfactory in his hands. We refer readers to the paper itself for the details. We will only say here, that as the old treatment of carbuncle, by every means we ever heard of, has been eminently unsatisfactory and unsuccessful, any plan that has met the expectation in the hands of so able and trained an observer as Dr. Osborn, should be hailed as a veritable boon. Carbuncle is a dreadful disease; and any remedy that will cure it, or mitigate its destructive and painful course, will be welcome.

Dr. Osborn has fulfilled the scriptural measure of years—having passed his three score and ten; and has been in the active practice of medicine for more than a half a century. Thoroughly educated in the principles of the science of medicine, he has been a careful observer at the bedside; and to his schooling has been added a course of reading embracing the whole scope of medical science. To these have been added the results of his observation and experience; the whole constituting a fund of knowledge of great value. He is, therefore, in position to speak *ex cathedra*, and his utterances are entitled to great weight and consideration.

The paper was read before the Johnson County Medical Society, and was highly complimented, especially by the younger members, who sit at the feet of the venerable preceptor. By unanimous vote it was sent to the Journal for publication.

